# Supplementary material for: Inferring Drug Set and Identifying the Mechanism of Drugs for PC3
Source: Int J Mol Sci. 2024 Jan 7;25(2):765. doi: 10.3390/ijms25020765 (PMC10815650; doi:10.3390/ijms25020765)
Supplement: Supplementary file 1 [file ijms-25-00765-s001.zip › ijms-2737251-supplementary.pdf]

Table S1. Unique drugs of each cluster from rank3

Drugs in cluster1	Drugs in cluster2	Drugs in cluster3
15-deltaprostaglandinJ2	deferoxamine	alpha-estradiol
17-allylamino-geldanamycin	fisetin	butein
celecoxib	genistein	butirosin
coppersulfate	Valproic acid	dimethyloxalylglycine
diclofenac	4,5-dianilinophthalimide	docosahexaenoicacidethylester
fasudil		estradiol
imatinib		fulvestrant
indometacin		mercaptopurine
LY-294002		monastrol
monorden		resveratrol
novobiocin		splitomicin
pirinixicacid		U0125
rofecoxib		HNMPA-(AM)3
rosiglitazone		
sodiumphenylbutyrate		
tetraethylenepentamine		
tretinoin		
trichostatinA		
troglitazone		
TTNPB		
Valproic acid		

Table S2 drug interaction in cluster2 in page9

	alpha-estradiol	diclofenac	estradiol	fulvestrant	genistein	imatinib	mercaptopurine
alpha-estradiol(ethinylestradiol)							
diclofenac	–						
estradiol	**+**	–					
fulvestrant	**+**	–					
genistein	–		–				
imatinib	–	–	–		–		
mercaptopurine		–	–			**+**	
resveratrol							**+**
rosiglitazone		–			–	–	
tetraethylenepentamine(colestipol)		–	–				
tretinoin		**+**	–			**+**	
valproicacid	**+**	–	**+**	–	–	**+**	–
